# Optimizing the role of ‘lead mothers’ in seasonal malaria chemoprevention (SMC) campaigns: formative research in Kano State, northern Nigeria

**DOI:** 10.1186/s12936-023-04447-z

**Published:** 2023-01-12

**Authors:** Ekechi Okereke, Helen Smith, Chibuzo Oguoma, Olusola Oresanya, Kolawole Maxwell, Chinedu Anikwe, Lawrence Chijioke Osuji, Obianuju Ogazi, Jamila Musa, Ashiru Rajab, Emmanuel Shekarau, Festus Okoh, Erica Viganò, Laura Donovan, Charlotte Ward, Kevin Baker

**Affiliations:** 1 Malaria Consortium, Abuja, Nigeria; 2 Independent Consultant, International Health Consulting Services Ltd, Merseyside, UK; 3 Kano State Ministry of Health, Kano, Kano State Nigeria; 4 National Malaria Elimination Programme, Abuja, Nigeria; 5 Federal Ministry of Health, Abuja, Nigeria; 6 Malaria Consortium, London, UK; 7 London School of Hygiene and Tropical Medicine, London, UK; 8 Department of Global Public Health, Karolinska Institute, Stockholm, Sweden

**Keywords:** Lead mothers, Seasonal malaria chemoprevention, Sustainability, Implementation research, Nigeria

## Abstract

**Background:**

Seasonal malaria chemoprevention (SMC) is a safe and effective intervention for preventing malaria in children under 5 years of age. Lead mothers are community health volunteers that help caregivers comply with monthly administration of anti-malarial drugs during SMC campaigns. The lead mother approach is used in several SMC implementing states across Nigeria, but there is lack of evidence about their roles and how effective they are. This study sought to better understand the current role of lead mothers, identify areas for improvement and ways to optimize the role of lead mothers during SMC campaigns.

**Methods:**

This paper reports the formative phase of a three-phased intervention development study. The formative phase involved semi-structured interviews with stakeholders from national, state, local government and community levels (n = 20). Thematic analysis was used to identify key themes, forming the basis of a subsequent co-design workshop with stakeholders routinely involved in SMC campaigns.

**Results:**

The findings of the formative phase converged around four overarching themes: skills and attributes required of lead mothers; factors that affect lead mother’s roles; how lead mothers interact with Community Health Influencers Promoters Services (CHIPS) agents and re-imagining the role of lead mothers during SMC campaigns.

**Conclusion:**

This formative work in Kano state indicates that through their strong connection to communities and unique relationship with caregivers, lead mothers can and do influence caregivers to adopt healthy behaviours during SMC campaigns. However, there is room for improvement in how they are recruited, trained and supervised. There is need to improve lead mothers’ knowledge and skills through adequate training and supporting materials, so they can deliver targeted health messages to caregivers. Sustainability of the lead mother approach is at risk if policymakers do not find a way of transitioning their role into the existing community health worker infrastructure, for example by using CHIPs agents, and ensuring less reliance on external donor support.

## Background

Most childhood deaths from malaria occur during the rainy season in areas of seasonal malaria transmission, for example in the Sahel region of sub-Saharan Africa [1]. In 2012, the World Health Organization (WHO) recommended seasonal malaria chemoprevention (SMC) for children aged between 3 and 59 months [2] to maintain therapeutic concentrations of anti-malarial drugs in the blood during the rainy season [3]. SMC is delivered in communities at household level, and involves the administration of full 3-day monthly courses of sulfadoxine–pyrimethamine (SP) and amodiaquine (AQ)(SPAQ), during peak malaria transmission [1]. SPAQ is administered through direct observation of SPAQ on day 1 and caregiver administration of two doses of AQ on days 2 and 3. However, a key challenge is ensuring adherence to the complete 3-day course of SPAQ—poor adherence could accelerate anti-malarial resistance and, therefore, affect the protective effect of SMC [4].

Sensitizing and engaging with communities before and during SMC delivery is a key part of ensuring the quality of SMC delivery. In Nigeria, community and religious leaders work collaboratively with key SMC personnel such as community drug distributors (CDDs), town announcers and lead mothers to deliver the annual SMC campaigns. *Lead mothers* (LMs) are female members of the community who are recruited by the SMC programme with the support of community leaders to conduct health promotion activities. They are important for increasing caregivers’ adherence to the administration of day 2 and 3 doses of AQ and ensuring that the complete dosages of SPAQ are administered during each monthly cycle. Lead mothers are renumerated for the number of months they work during SMC campaigns by the SMC programme. Because lead mothers are females and well known by community members, they can enter caregivers’ homes and establish an interpersonal relationship with caregivers; this also provides an opportunity for caregivers to have their questions answered and concerns addressed.

Within the SMC programme in Nigeria, lead mothers are selected based on the following criteria: age (at least 18 years); previous experience (in mass drug administration, house to house vaccination campaigns or health promotion activities); residence (must reside in the community (or ward) where she is assigned to work); availability (should be available for the 4-day period of SMC delivery during the monthly SMC cycles and not involved in any other healthcare intervention during the SMC cycles). Further to this, a lead mother should be recommended by the community leader of the community/ward where she will be assigned to work; as part of the recommendation the community leader should provide a good character reference. Finally, each lead mother must complete and submit a biodata form with a valid photo identification, should have a functional phone number as well as a valid bank account (to receive monthly renumerations).

In the published literature, the LM model appears to vary in different contexts with respect to their roles in the community and linkages to the health system. In Sri Lanka, an established volunteer LM programme focuses on strengthening mothers’ engagement in child health, nutrition and development through strong community participation [5]. Here, LMs act as peer educators to improve the knowledge, attitudes and practices of primary caregivers on infant and child nutrition through the formation of discussion groups. Similar approaches exist elsewhere, such as in Sierra Leone where LMs facilitate mother care support groups and conduct home visits to provide relevant information on topics such as breastfeeding and nutrition [6]. In Uganda, there has been a LM programme, focused on improving nutrition and feeding practices, where LMs work in collaboration with village health teams and the local health centre [7].

In many countries (including Nigeria) the presence of various stakeholder-driven community-based health worker programmes have led to considerable verticalization of community health structures, poor coordination, inefficiencies and concerns about value for money [8]. Most of these programmes are similar in context and concept but are not linked in a manner that enables the various programmes to leverage existing structures for the attainment of common goals and objectives. In response, a Community Health Influencers Promoters and Services (CHIPS) Programme was designed in Nigeria, established by the National Primary Healthcare Development Agency (NPHCDA) in 2018 [9, 10] and endorsed by the Federal Government of Nigeria. The aim of the CHIPS Programme is to improve demand for, access to, and equitable coverage of essential health services, especially those related to maternal and child health at the community level. CHIPS agents are responsible for community-based health education and demand creation for health services through community mobilization, home visits and referrals. The CHIPS programme is funded via the federal government, with state governments being responsible for programme planning and implementation [10]. A few states in Nigeria have adopted the CHIPS strategy and in Kano state, the programme is implemented through the Kano state Primary Health Care Management Board (KSPHCMB) in 10 local government areas (LGAs). The programme is in its first phase of implementation in Kano state, with plans of scaling up to more LGAs in subsequent years.

Although LMs have been working for some years within communities in Nigeria to help support adherence to anti-malarials and malaria prevention measures in LGAs where SMC is implemented, there is lack of clarity about their role and impact. It is also unclear how LMs interact with other key community-level actors, such as the CHIPS agents. Malaria Consortium, working with the National Malaria Elimination Programme in Nigeria, Kano State Ministry of Health and Kano state Primary Health Care Management Board designed a three-phased intervention development study to optimize the role of lead mothers in one LGA in Kano state.

This paper reports findings from the formative research, carried out to better understand the current role of lead mothers, identify areas for improvement and ways to optimize the role of LMs in order to improve health outcomes.

## Methods

### Study setting

Kano state is in north-western Nigeria and has a population of over 12 million people. Hausa and Fulani are the most prominent ethnic groups and Hausa language is the main language in the state. Similar to other northern Nigerian states, malaria is endemic in Kano state with all year-round transmission, but seasonal peaks occur during the rainy season. Kano has two seasonal periods per year—about 5 months of rainy season (May to September/October) and 7 months of dry season (October/November to April) [11].

SMC campaigns started in Kano state in 2016, and are currently implemented by Malaria Consortium via the Global Fund Malaria project. Minjibir LGA was selected as the study site because it is one of the ten LGAs where the CHIPS programme is being implemented alongside SMC campaigns, where LMs are employed and where adherence to anti-malarial doses on days 2 and 3 is lower compared with other LGAs in Kano state. The LGA recorded the lowest adherence figures for days 2 and 3 during the end of round survey for the 2020 SMC campaigns, among LGAs where the CHIPS programme is being implemented. The LGA’s proximity to Kano metropolis and ease of access as well as the relatively stable security situation were additional factors considered when selecting the study LGA.

### Study design

This formative research is part of a larger intervention development study that consists of a subsequent design phase involving stakeholders in co-designing an intervention to optimize the role of LMs and pilot testing it; plus, an evaluation phase to assess feasibility, acceptability, and implementation factors. The formative research involved semi-structured interviews with stakeholders from all levels of the health system (community, LGA, state and national levels).

### Conceptual framework

**Fig. 1:**
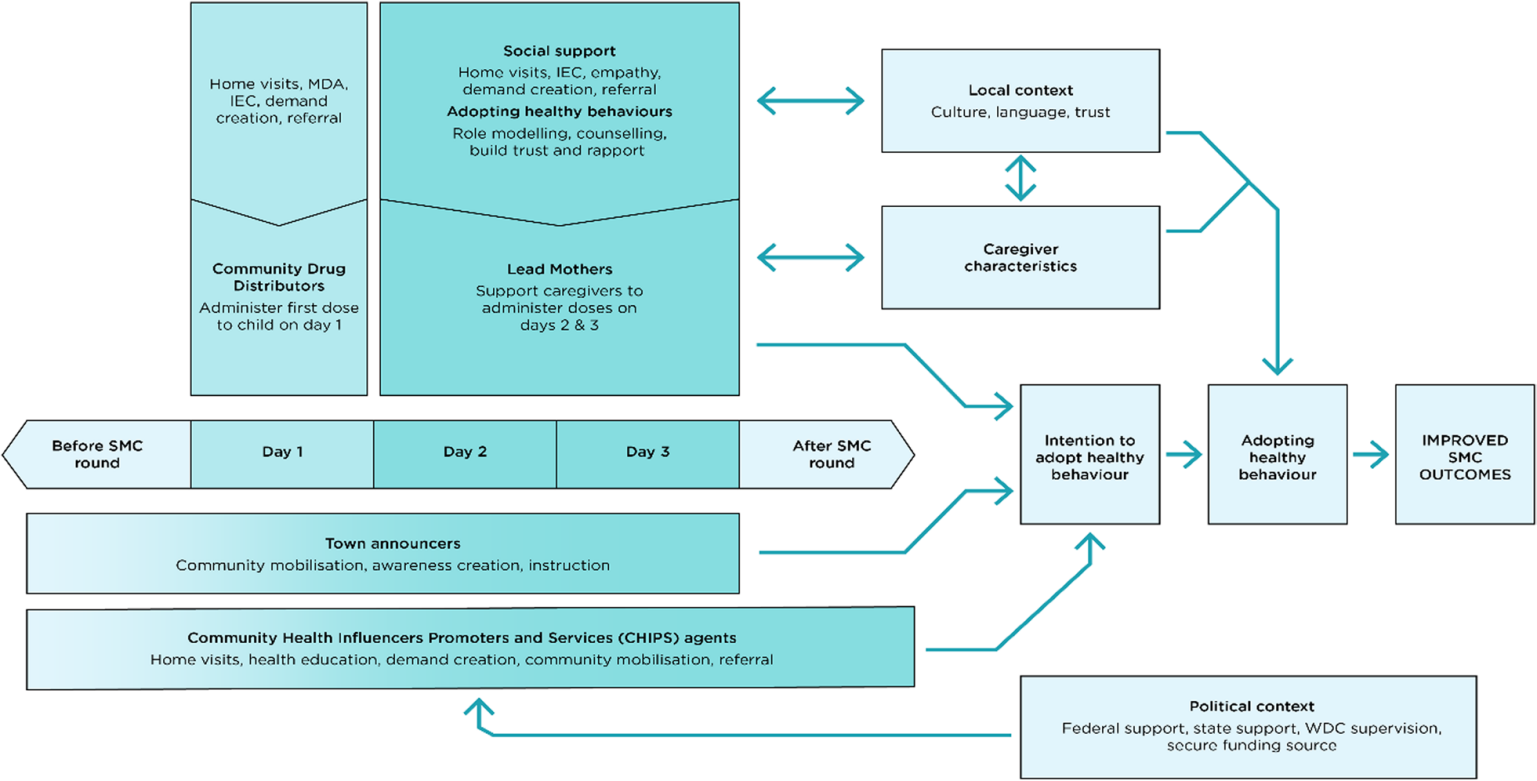
Conceptual framework for the study

This study conceptualized the relationship between various community-level actors and how they contribute to SMC delivery at LGA level in Nigeria in Fig. 1. This is adapted from an existing framework that explains how community health workers (CHWs) facilitate adoption of healthy behaviours by community members, by working together as ‘partners in health’ [12]. In the study’s framework, the key community actors in SMC campaigns and community-level healthcare interventions (town announcers, community drug distributors, lead mothers, CHIPS agents) are indicated as well as when during SMC campaigns they contribute and what their key roles are. The figure also highlights the influence of the political context, local context and caregiver characteristics on these actors and their roles, and how these can affect SMC programme outcomes. For example, caregivers’ characteristics (including age, parity, education level) can directly influence how LMs are able to interact with caregivers and fulfil their roles. Additionally, the ‘local context’ (especially culture and language) can influence the extent of social support and assistance LMs are able to provide to caregivers, with possible consequences for SMC programme outcomes. The ‘political context’ including policy and financial support from the Federal and State government infrastructure for planning and implementation, can influence the effectiveness of the CHIPS programme, but can also impact on the work of the LMs and CDDs during SMC campaigns. There is more reference to this framework when interpreting the findings from the formative research.

### Sampling and data collection

A desk-based mapping exercise was conducted to identify the most appropriate key informants. The research team listed all stakeholder organizations involved in SMC campaign planning and implementation in Kano state, as well as individuals at LGA and community level actively involved in SMC delivery. The list was reviewed with representatives from the National Malaria Elimination Programme, the Kano state Ministry of Health and the Health Department of the study LGA, to identify ‘information rich’ individuals within each stakeholder group to invite for interview. Because of the interest to assess the relationship between CHIPS agents and lead mothers, CHIPS agents and their supervisors were included in the list of study participants.

Key informants were identified based on purposive selection criteria, with the aim of achieving a sample of ‘information rich’ individuals that was balanced and diverse enough for comparisons to be made during analysis. The main criteria were health system level (community, LGA, state and national level) and organization type (directly involved in the planning or implementation of the SMC campaign, aligned community health programmes). Other criteria such as key informant experience (decision-makers with considerable experience working in SMC or aligned health programmes) and gender (men and women), were monitored as recruitment progressed to ensure a mix of men and women with appropriate experience. Using principles for deciding saturation [13], the initial study sample was a minimum of 16 key informants (approximately n = 4 from each health system level) and the study team estimated an additional one or two interviews may be needed at each level until no new ideas emerged (the stopping criterion). After each successive interview the research team reviewed field notes to judge if data saturation had been reached and data collection could stop.

Potential participants at national and state levels were first contacted by the research team by email, which included an invitation to participate and the study’s ‘participant information sheet’. After 1 week, the study team followed up those invited by phone call to determine their interest, answer any questions and arrange an interview date and time for those who agreed to participate. Potential participants at community and LGA level were contacted by phone and invited to participate in the study by the research team, the content of the participant information sheet was provided verbally and individuals were given time to consider participating in the study. For the individuals who agreed to participate, suitable dates and time were agreed with the research team. Semi-structured interviews were held in September and October 2021; state and national level key informants were interviewed by telephone calls or face to face in their offices. Participants at community and LGA levels were interviewed at the offices of the LGA Health Department in the study LGA. Each interview was conducted by two (trained and experienced) research assistants, who audio-recorded and made brief field notes during and immediately after each interview. Interviews were held in English except for those at community level, most of which had their interviews conducted in Hausa Language. Topic guides covered key questions relating to the overall study purpose; questions were broad and open ended with prompts to explore the following: the role of lead mothers, how they influence caregivers, their relationship with other community health workers (especially the CHIPs agents) and areas for improvement. Separate topic guides were adapted for use with key informants at each level. Interviews lasted for between 45 min and 1 h.

### Data analysis

Interviews at community level were translated and all interviews from the different levels of the health systems were subsequently transcribed. Audio files were transcribed verbatim by the same research assistants. Data were analysed using the ‘codebook’ approach to thematic analysis [14]. Initial coding lists were developed by the research assistants based on the topic guides used with key informants at each level. The code lists were reviewed by the first and second authors of this paper, who suggested additional codes based on a review of selected transcripts. Revised coding lists were discussed and agreed by the whole team. The research assistants then used the agreed code lists to code all transcripts manually in MS Word; further inductively derived codes were added as coding progressed. Coded data were further interrogated, and related codes collated into preliminary themes and sub-themes across each level. Preliminary themes were reviewed and discussed in whole team meetings with the research assistants and other research team members to ensure consistency and meaning. Once refined and agreed, each theme was described, and comparisons made between perspectives at each health system level. Table 1 shows how coded extracts link to sub-themes and overarching main themes.

**Table 1:** Overview of codes, sub-themes and overarching themes

Codes	Sub-themes	Overarching themes
Shared customs/religion/beliefs, familiarity with environment, communal acceptance	Familiarity with communities	Skills and attributes required of lead mothers
Effective communication, manner of approach	Communication skills	
Political interference, age, lead mothers are insufficient	Recruitment of lead mothers	Factors that affect lead mother’s roles
Inadequate training, inadequate knowledge of job description	Training of lead mothers	
Poor supervision, absence of workplan	Work planning and supervision	
Delayed payment of stipends, increase stipends	Remuneration and delayed payments	
Differences in role of lead mother and CHIPS agents	Distinct roles	How lead mothers interact with CHIPS agents
Similar role of lead mother and CHIPS agents	Harmonized roles	
Political and financial support for CHIPS agents	Sustainable capacity	
CHIPS agents’ involvement in SMC, increased workload	A supporting role for CHIPS agents	Re-imagining the role of lead mothers
Strengthen criteria for selection	Review and strengthen selection criteria	
Review current supervision guidelines,	Strengthen supervision	
Capacity building, interpersonal communication skills	Targeted and tailored training	
Health promotion, integration, increased access to care	Integrate health promotion and prevention	
Sustainability, government policy and funding support	Sustaining programme capacity	

## Results

Twenty key informant interviews were conducted across the four health system levels (community, LGA, state and national); study participant characteristics are outlined in Table 2. The key informants for the formative research were from across National (n = 2), State (n = 6), LGA (n = 6) and community health system levels (n = 6). There were representatives from the National Malaria Elimination Programme, Kano State Malaria Elimination Programme, Kano State Ministry of Health, Minjibir LGA Health Department officials, Minjibir LGA Health facility in-charges, CHIPs agents, CHIPs supervisors as well as community leaders. Broadly, their job roles include government officials/policy makers, health workers and community leaders/community health volunteers. There were 14 and 6 male and female key informants respectively who participated in this formative research.

**Table 2:** Descriptive characteristics of study participants

Variable	Qualitative sample (N = 20)
Health system level	
National level	2
State level	6
LGA level	6
Community level	6
Organization	
National Malaria Elimination Programme	2
Kano State Malaria Elimination Programme	2
Kano State Ministry of Health/Kano State Primary Health Care Management Board	4
Minjibir LGA Health Department officials	4
Minjibir LGA Health facility in-charges	2
CHIPS agents	2
CHIPS supervisors	2
Community leaders	2
Job role of key informant	
Government officials/Policy makers	8
Health workers	6
Community leaders/Community health volunteers (e.g. CHIPS agents, CHIPS supervisor)	6
Sex of key informant	
Male	14
Female	6

The findings of the formative work converged around four overarching themes: *skills and attributes required of lead mothers*; *factors that affect lead mothers’ roles*; *how lead mothers interact with CHIPS agents*; and *re-imagining the role of lead mothers in SMC campaigns*. Each theme is described below, with illustrative quotes.

### Skills and attributes required of lead mothers

Key informants at each health system level highlighted the attributes and skills of LMs that help them to establish good relationships with, provide support to and influence the behaviour of caregivers during the SMC campaigns. Shared customs and beliefs as well as being known in the communities helped lead mothers engage with caregivers. Being good communicators and knowledgeable about health promotion were regarded as valuable skills to have as lead mothers.

### Familiarity with communities

Many key informants indicated that lead mothers are usually familiar with the communities in which they work, and are supposed to be recruited from within these communities. It was reported that LMs typically know the terrain making it easier for them to access households during SMC campaigns, including those in hard-to-reach communities. It was pointed out that communal acceptance of the work of LMs is also key to caregivers accepting SPAQ during SMC campaigns.

*“…those persons (LMs) are people from the community, that’s why we emphasize their selection to be from there, so that they are not seen as strangers.”* (National level health official).

*“They are people close to the community, [so the] community can find it very easy to understand or accept whatever they bring to them, that is one of the advantages.”* (State level health official).

Being from the same community also means lead mothers are perceived as sharing customs, norms, beliefs and religion with the households they visit. State level officials believed this was important as it allowed LMs to approach, engage and communicate with caregivers during SMC campaigns. They felt this helped LMs to influence the health behaviours of caregivers’ and their attitudes towards SMC.

…they are very familiar with the community…even the norms and culture of the communities.

(State level health official).

### Communication skills and manner

Many key informants identified that in principle, effective communication and the manner of approach were important in enabling LMs to influence caregivers’ behaviour. State level officials felt that interpersonal skills were the ‘backbone’ of effective community health services. Many of those interviewed mentioned that, if effective and positive, communication and conduct of LMs would ensure that the complete dosages of SMC medicines are administered during SMC campaigns. However, some participants indicated that LMs should be trained to acquire effective communication skills and informed about why their manner with caregivers is important.

*“…creation of rapport is the backbone of almost everything, bringing good relationship between you and the caregiver, if that is done well, whatever you come with, they will really accept you.”* (State level health official).

*“…the way you interact with people, that is what would make them to accept you whenever you come up with something.”* (National level health official).

While these are generally within the tasks of CDDs, key informants at LGA level believed that LMs have a critical role in supporting heath education and promotion, especially the need to demonstrate proper drug administration to caregivers and explain the importance of completing the 3-day course of anti-malarials. Key informants at LGA level believed that a critical role for LMs was demonstrating to caregivers exactly how to dissolve the tablets in water and helping the child to drink the solution. State level officials emphasized the role of LMs in identifying fever and symptoms of malaria as well as providing care-seeking advice to caregivers.

*“…they are helping them through demonstration because we give them training on how to instruct caregivers and mothers at home, how to make sure that drugs have been diluted and given.”* (LGA level health official).

*“…if the child is not feeling ok, like if he has fever, they would tell them to go to the health facility; I know the lead mothers can identify the signs of illness and ask the caregiver to take the child to the hospital so that the child can go through the diagnosis process.”* (State level health official).

### Factors that affect the role of lead mothers during SMC campaigns

Key informants from national, state, LGA and community levels identified several impediments to LMs carrying out their roles. Key aspects mentioned were recruitment, training, work planning and supervision, as well as remuneration and delayed payments. They argued that if these factors were addressed, LMs could better perform their roles within SMC campaigns.

### Recruitment of lead mothers

Most key informants from all health system levels stated that the current approach of recruiting LMs annually to support SMC campaigns is disruptive—LMs recruited in 1 year may not be recruited the following year which invariably affects sustainability of the approach. The fact that recruiting takes place every year results in some LMs being dropped and sometimes unreasonably, for instance due to political interference from ‘powerful actors’ within the state’s health system.

*“…changing of lead mothers affect the program, because the more someone is doing the job, the more experienced they will be.”* (State level health official).

Based on standard guidelines, community leaders are expected to nominate LMs from within their communities, however many key informants indicated that there is a lot of political interference in the selection process. This results in some LMs being selected without the necessary qualifications or who are not from the communities, which invariably affects the performance of the lead mothers.

*“…majority of the candidates of politicians refuse to do their job…but the remaining who were selected based on merit, they do their job.”* (LGA level health official).

The SMC guidelines stipulate that LMs should be at least 18 years old. Most key informants felt it was important that this minimum age limit be adhered to, as it ensures LM can communicate effectively with caregivers and convey important health messages during SMC campaigns. Some also argued that older women should be considered for LM roles as they have more experience. State level officials argued that LMs need experience as a mother themselves, so caregivers will be more receptive to their health messages and pay more attention to the LM.

*“…Some of the gaps is that you will find a lead mother who herself has never delivered any child for instance…That’s why I said that they should be matured.”* (State level health official).

A common view was that there were insufficient LMs being recruited, and this has affected the coverage of households in LM-assigned communities during SMC campaigns. The current ratio of one LM to cover the households visited by five CDD teams was described as inadequate (each CDD team is expected to visit up to 70 households per day). Many key informants felt this was unrealistic and prevented LMs from meeting their daily targets. Some key informants at State and LGA levels explained that lead mothers tend to pick a sample of households to visit each day, rather than their overall target number, and this results in inadequate support to caregivers to adhere to the 3-day course of anti-malarials for their children.

*“I’ve talked about the fact that they are inadequate (in numbers) from my own perspective…and so they need to have more of them selected…more of them is required to optimize their role.”* (National level health official).

*“…the lead mothers are not enough…honestly speaking they are not enough to cover their expected target. This is one of the problems that we are experiencing”* (State level health official).

*“They should be going to all the settlements to enlighten people on the importance of the antimalarial drug for children*” (Community leader).

### Training of lead mothers

Many key informants from the different levels of the health system indicated that training for LMs is inadequate and this means they lack the requisite skills to function in their role. As well as training being too short, key informants at LGA level argued the content should be reviewed; LMs should have their own tailored training, probably delivered to them separately from other SMC campaign staff, to ensure that they properly understand and then adequately carry out their role.

*“…let them be well educated, well trained and well equipped on how to mobilise, educate people (even the mothers) who are giving the drugs at home, how to give the drugs and the importance of the drugs, before they should be given this work.”* (LGA level health official).

“…*make sure that lead mothers are given more training…let them be trained separately*.” (LGA level health official).

### Work planning and supervision

Across the health system levels, key informants complained about poor supervision during SMC campaigns. There were suggestions that some supervisors, selected for SMC campaigns, had no real interest in conducting supervision for SMC campaigns, because the selection process is not based on merit. Key informants from LGA and community levels described how some supervisors are approached and appointed because of their relationships with influential key actors involved in SMC campaigns within the state, so there is no commitment on their part to perform their role effectively. It was reported that many supervisors do not actually go to the field for supervision and thus do not provide the required support to LMs.

*“…supervising the lead mothers will improve their work during SMC campaigns because if you are not supervising them, some will not go round to all the houses assigned to them and they will come and say they have finished doing their work but if you are (properly) supervising them, they will definitely visit all the houses.”* (Community level health official).

*“…[effectively] supervising them can improve them.”* (State level health official).

State-level key informants also implied that many LMs fail to properly understand their role, and it is therefore unfair to expect them to perform well. Some state level health officials argued that many LMs work without workplans to guide them during SMC campaigns.

*“…most of the lead mothers…honestly, they don’t even know their work…as I have said and I will still repeat, most of them don’t even know what is expected of them, so there is a problem with that.”* (State level health official).

*“…a lot of the lead mothers don’t have workplan”* (State level health official).

### Remuneration and delayed payments

There were many complaints about delays in the payment of stipends to community level SMC staff, including LMs. Key informants at all levels argued that delayed and inadequate payment undoubtedly affects the ability of LMs to carry out their role during SMC campaigns. Some mentioned that the stipend paid to LMs is insufficient considering the number of households they are meant to visit. This was thought to have serious consequences, especially for their work in hard-to-reach communities.

*“…there is delay in payment…you know if you pay them early, you motivate them in order to work effectively…but if there is a delay in payment, there is no motivation for them to work effectively.”* (State level health official).

*“…the stipends provided for them to transport themselves is small, so [this] contributes to their inability to actually meet the target for which they are assigned to meet daily.”* (National level health official).

### How lead mothers interact with CHIPS agents

This theme describes how lead mothers and CHIPS agents interact and makes clear the similarities and differences in their roles in Kano state. There were calls from LGA, state and national level key informants for CHIPS agents to be integrated to work during SMC campaigns, to support and complement the work of lead mothers.

### Distinct roles

Key informants at all levels agreed that both LM and CHIPS agents are community-based health volunteers/workers involved in health promotion activities. While LMs are recruited specifically to support adherence to anti-malarials during SMC campaigns, CHIPS agents have a broader health promotion remit for maternal and child health.

*“CHIPS agents and lead mothers are quite different…because CHIPS agents have been selected by National Primary Health Care Development Agency (NPHCDA) to conduct all healthcare services including maternal and child health, malaria prevention, diarrhoea disease control, pneumonia disease control and other health education…whereas lead mothers are selected based on SMC programs to make sure antimalarial drugs are given to children for the second and third days.”* (LGA level health official).

*“…the role of the lead mother is to mobilise and ensure that she explains to caregivers about SPAQ, only SPAQ [during SMC campaigns]. While CHIPS agents are there always and she talks about any area. She talks about health improvement, their environment, their foods, children [and taking them to the hospital], antenatal care, immunization and so on…”* (Health facility in-charge, Minjibir LGA).

### Harmonized roles

Many key informants felt that CHIPS agents should be integrated with SMC campaigns and that they could contribute to the work of LMs. For example, a community level health official stated that following up with caregivers in the community is already the work of CHIPS agents and that working together with LMs will give a very good result.

*“…CHIPS agents do health promotion and that is also what the lead mothers do basically…health promotion by the lead mothers is basically on malaria but health promotion by CHIPS agents is on health more generally.”* (National level health official).

*“…follow up is already the work of CHIPS agents and working together with lead mothers will give a very good result.”* (CHIPS agent).

*“…all CHIPS agents are already known in the community like the lead mothers and are familiar with the people of the community, people trust both lead mothers and CHIPS agents*.” (CHIPS supervisors).

### Sustainable capacity

It was also argued that involving CHIPS agents in SMC campaigns would promote sustainability, since the CHIPS programme enjoys state level political and financial support, while the intervention of LMs (just like much of the SMC programme) is reliant on donor organizations. Key informants at all levels believed that introducing CHIPS agents to support LMs would boost capacity and help build sustainability in the SMC programme.

*“It is very important to involve CHIPS agents in SMC program because they have more capacity than the lead mothers in terms of qualifications, experience and other things that will help SMC program to be successful.”* (Community leader, Minjibir LGA).

### Re-imagining the role of lead mothers in SMC

When asked what an ideal lead mothers programme would look like, key informants at each level reconsidered current LM roles and suggested improvements or modifications, resulting in a re-imagined role for LMs and CHIPS agents in state level SMC campaigns. Many suggestions are based on the challenges reported in other themes.

### A supporting role for CHIPS agents

A widely discussed suggestion was the need for CHIPS agents to be involved in SMC campaigns to support the role of LMs. Some LGA-level key informants suggested that CHIPS agents should be involved as supervisors while others at state-level felt they would be better in a supportive role helping LMs to ‘follow up’ on households on days 2 and 3 during SMC campaigns. It was argued that involving CHIPS agents to follow-up with caregivers would add value and address the shortage of LMs to carry out this role. Across all health system levels, key informants acknowledged that CHIPS agents had more capacity, experience and were better trained to deliver a range of health messages [beyond malaria prevention and SMC] to caregivers and community members. A minority of stakeholders were cautious, stating that the increased workload for CHIPS agents may invariably affect the quality of their work.

*“…CHIPS agents should be the supervisors of the lead mothers…that should be better.”* (LGA level health official).

*“…we can use CHIPs agents for subsequent SMC campaigns to carry out the role of lead mothers, I think it is a good idea.”* (State level health official).

*“…employing more people will be better, but not CHIPS agents because the workload will be much on them.”* (Community leader, Minjibir LGA).

### Review and strengthen selection criteria for lead mothers and supervisors

A clear recommendation from many key informants was for selection and recruitment processes to be reviewed and strengthened. It was argued that there was a need to improve processes to ensure the right people were selected, on merit, for LM and supervisor roles. Others suggested minimum requirements and qualifications for LM roles, for example, state level officials advised that LMs should be literate in order to be able to understand the key health messages to be disseminated to caregivers.

*“…improve the quality of the lead mothers right from recruitment, because that is why we can see some gaps as we have currently.”* (State level health official).

*“…I would want to see a period of screening first, even before invitation for training. I think that will improve the quality of the lead mothers.”* (State level health official).

### Strengthen supervision of lead mothers

Key informants at national, state, LGA and community levels stressed that the supervision of LMs is currently weak. A dominant view was that strengthening supervision should begin with more rigorous recruitment practices to avoid recruitment of those connected to influential figures in the state health system. Key informants revealed inadequacies in the current supervisory structure, where one supervisor oversees CDD teams and LMs at the same time. One LGA level health official stressed the need for stronger supervision and demanded one supervisor per LM or a group of LMs. Further to this, to overcome weaknesses in supervision and spot-checking of LMs, a review of current supervision guidelines was suggested as a way to make more visible the role of supervisors, and to emphasize aspects of their role during training.

*“…when lead mothers are back from the field and they present their daily record of work, there should be people who will go for spot checking to visit two to three houses in every settlement the lead mothers visited the previous day; this is to make sure that the lead mothers do their job right every day and meet up their targets.”* (Community level health official).

*“…there is need for supervisors for the lead mothers themselves, because joining the supervisors of CDDs and lead mothers, really gives problems.”* (State level health official).

### Targeted and tailored training is required

Many key informants emphasized the need for more and better training for LMs to improve their performance. Gaps identified include inadequate knowledge of malaria prevention and SMC as well as inability to respond to caregivers’ and family members’ questions. At community level, the need for refresher training at the beginning of each cycle was reiterated. State level health officials highlighted that caregivers and community members would be more receptive if they perceived that LMs were knowledgeable and communicated health messages convincingly when they visited communities. They recommended incorporating interpersonal communication skills and how to build rapport with caregivers into training for LMs.

*“…how can you give someone an assignment without [proper] training, you have to tell the person how to do it, how to deliver it and how to enter the community, yes training is very important. The training given to the lead mothers; I don’t think it is enough for them to even know their roles…they need more training.”* (State level health official).

*“Before commencing every cycle, it is important that they should have a refresher course. Or on the job training. To teach them how to deliver their work. Because most of them their level of education is very low.”* (Community leader, Minjibir LGA).

*“…if the lead mother has the proper knowledge and appropriate information to pass to the caregivers, more often than not, the community or the mothers will listen to them very well.”* (State level health official).

### Integrate health promotion and prevention activities

Some key informants at national level highlighted the need for lead mothers to support the integration of malaria interventions within the community. For example, lead mothers could promote other malaria prevention strategies, including the use of insecticide-treated nets, when they visit households during SMC campaigns. Many key informants were aware that SMC alone is insufficient and other preventive measures are needed to protect vulnerable groups from malaria.

*“…we emphasize the fact that SPAQ alone will not confirm 100% protection on the children, so the need to ensure their deployment alongside other interventions…for instance long lasting insecticide treated nets”* (National level health official).

*“…look at the household generally, the level of cleanliness whether they are using insecticide treated nets in the household or not, whether they have dustbins filled with trash where mosquitoes are hiding… [lead mothers] can be mobilized for preventive measures against malaria i.e., environmental sanitation, clearing of grasses, avoiding stagnant water in our communities.”* (State-level health official).

State and LGA-level health officials were emphatic that LMs could do more. They suggested LMs could be trained to deliver targeted messages to caregivers on improving sanitation, promoting better nutrition practices especially for children, as well as encouraging the use of other malaria prevention interventions among caregivers. There were also suggestions that LMs be provided with flipbooks, conveying important health messages through pictures, for use during their visits to households.

*“I can see the need for lead mothers even outside malaria chemoprevention. We can use them to impact knowledge on the community on many health aspects. Nutrition, for example, as they are going in to do malaria chemoprevention, they are also looking at the children”* (State-level health official).

*“…they [can] give advice on personal hygiene, maternal and child health services…and they can also make sure that referrals have been made.”* (LGA level health official).

*“I suggest these lead mothers should have pictorial flipbooks that show healthy living messages in [proper] order, this will help lead mothers when they see and counsel the caregivers…”* (State level health official).

### Sustaining programme capacity

Many key informants stressed the need to address sustainability of the SMC programme, calling for a ‘sustainable structure’ that can be called upon at any time to deliver the SMC programme. A national level health official stressed that to improve sustainability, the SMC programme should actively work with existing community health system structures, including the ward development committees. Many key informants also raised the point that the current SMC intervention is almost completely supported by donor organizations; and if funding is withdrawn, this would significantly affect implementation of the SMC programme in Nigeria. Many suggested that introducing CHIPS agents to support the role of LMs might help sustain capacity in the programme. The CHIPS programme has government policy and funding support, while the current LMs intervention (and the SMC programme more generally) is supported every year mainly by donor organizations. Some stated the importance of reducing the disruptions which arise from the annual recruitment of LMs (and other community level personnel) because such changes affect the sustainability of the SMC programme.

*“The lead mothers who worked in 2020, if you want to assess how many of them are doing 2021 work, you will see a huge difference.”* (State level health official).

*“…the most serious challenge is even when you have change in political setting. You will see different set of lead mothers. The constant recruitment of lead mothers affects the quality.”* (State level health official).

## Discussion

This formative research, part of a larger study to optimize the role of LMs in SMC campaigns, has helped to better understand the role LMs play, how they influence caregiver behaviour, and identified areas for improvement. Interviews were conducted with key informants from all levels of the health system, while focusing on one LGA in Kano State where CHIPS agents currently work and SMC campaigns are held annually. Findings reveal the skills and attributes required of LMs and factors that affect their roles, highlighting how LMs interact with CHIPS agents and how the LM strategy within the SMC programme can be improved.

The premise of the LM strategy is that females from within communities possess a unique set of skills, have the ability to interact with fellow community members, can easily access households and communicate effectively with caregivers. In line with the study’s conceptual framework, key informants outlined how the characteristics of LMs and local contextual factors affect the extent of support and assistance LMs can provide. However, the study findings identified additional factors that have an important influence on the roles of LMs. For example, the annual recruitment process causes disruption and poses risks to sustainability of the strategy. Concerns about annual recruitment of LMs are similar to those raised in a study of retention of community-based SMC staff in Burkina Faso [15]. It is recognized that when staff are retained and able to work in successive campaigns, they have a better understanding of their role and this is something future SMC campaigns in Kano state and other areas where SMC campaigns are implemented should aim for.

Further to this, current recruitment strategies were criticized for sometimes failing to select local females with the right characteristics to work as LMs, and the findings highlight a need to review recruitment and selection processes. A main concern among key informants was political interference, which led to LMs (and supervisors) being selected based on personal contacts rather than on merit. Such political interference undermines proper accountability in community health programmes [16]. To counteract this, high-level advocacy to relevant stakeholders is recommended before and during the selection process to ensure that minimum selection criteria are widely known. A further suggestion is to actively involve ward development committees and LGA health teams in screening and selecting lead mothers from within their own localities. The suggestion to recruit more older women, who have children and the experience of motherhood, may well improve relations between caregivers and LMs, probably resulting in better acceptance of the support and assistance which LMs provide to caregivers during SMC campaigns. One way to achieve this would be to set a new minimum age limit of 20 years for LMs, in line with the current guidelines used for recruiting CHIPS agents.

The overlap in the health promotion roles of LMs and CHIPS agents was recognized by stakeholders at all levels in this study. Although CHIPS agents have a broader health promotion remit, much of their work involves malaria prevention while LMs are recruited specifically to work during the SMC campaign to support adherence to anti-malarials among eligible children and promote malaria prevention measures. A dominant view was that CHIPS agents have more capacity, experience and were better trained than LMs and should be more involved in SMC campaigns. Suggestions included giving them a supportive role alongside LMs or engaging them as the supervisors of LMs during SMC campaigns. Key informants argued that this would strengthen the work of LMs and improve the quality and breadth of health messages delivered to caregivers. Key stakeholders identified that integrating CHIPS agents in SMC campaigns would also help build a more sustainable structure to support the SMC programme. In contrast to the LM strategy, which is precariously dependent on short-term donor funding, the CHIPS programme is endorsed and financed by the Federal government. Much more needs to be done by the Federal Ministry of Health in partnership with the National Primary Healthcare Development Agency (NPHCDA) and the National Malaria Elimination Programme (NMEP) to support the SMC programme, working collaboratively with the same agencies at state level to ensure sustainability of annual SMC campaigns, while building on and utilizing sustainability frameworks [17, 18].

Feedback from key informants indicated that when LMs established empathy, trust and rapport with caregivers they were able to assist and support them to adopt healthy behaviours, as implied in the study’s conceptual framework. However, stakeholders identified some obvious gaps in the communication skills of LMs and their ability to build rapport with caregivers, and suggested more targeted training, tailored to their needs. Recent WHO guidelines on health policy and system support for community health worker (CHW) programmes mandates that the scope and type of CHW training should reflect the kinds of roles they perform [19]. Suggestions from the study findings include incorporating effective communication and strategic behavioural change communication (SBCC) skills to increase the credibility of LMs in imparting health messages. WHO guidelines reiterate the importance of interpersonal skills related to community engagement and mobilization for CHWs to effectively perform in their role [20]; and key informants in this study felt that this should form a critical part of the training of LMs. Furthermore, on completion of training, LMs should properly understand their job role, including how to encourage caregivers to administer the correct dosage of SPAQ, why this is important and how to explain the protective effect of SMC for vulnerable children. More could be done to support LMs to communicate health messages more clearly, including by providing them with pictorial flipbooks to use during visits with caregivers. Lead mothers (and their supervisors) should be trained on flipbooks and any additional tools introduced for their use during SMC campaigns. Evidence from Gambia suggests that pictorial treatment cards can help support community level SMC personnel with low literacy levels [21].

As with any community-based health programme, there are always calls from health officials for CHWs to do more. In this study, stakeholders identified a range of additional roles for LMs, including advocating for healthy nutrition for children, encouraging better sanitation practices within communities, discussing other malaria prevention interventions including the use of insecticide-treated (ITNs) as well as promoting better health-seeking behaviour. While all of these activities could undoubtedly improve community awareness and positively influence health behaviours, it is unclear whether LMs have the capacity to absorb these additional tasks. As argued by others, there should be a careful consideration of the practical implications of introducing additional roles for community health workers and volunteers, including concerns about workload, costs and training requirements [22]. Programme planners and policy makers need to discuss with key stakeholders about the feasibility and best modalities for introducing these new roles for LMs within SMC campaigns, especially since previous research cautions against adding new roles for community level SMC staff [15].

There is evidence that delayed and inadequate payments to LMs, like for most CHWs, affects their ability to perform their roles, and motivation to work [23]. Such complaints need to be addressed, especially since these delays in payment may affect all community level SMC staff, not just lead mothers. There should be further consideration given to increasing the stipends for LMs, especially those who work in hard-to-reach communities, who face additional costs to reach those communities during SMC campaigns.

## Study limitations

The findings from this formative research and its implications are perhaps generalizable to other LGAs in Nigeria, where SMC campaigns are implemented and CHIPS agents currently work. However, the local context in each SMC implementing LGA, state or area needs consideration and there may be nuances in how SMC campaigns operate as well as how different community volunteers and CHWs are involved. In addition, some key national level key informants who were contacted to participate in the formative research, eventually did not take part in the study.

## Conclusion

This formative work in Kano state indicates that through their strong connection to communities and unique relationship with caregivers, LMs can and do influence caregivers to adopt healthy malaria prevention behaviours during SMC campaigns. However, there is a need to improve the knowledge and skills of LMs through adequate training and supporting materials, so they can deliver targeted health messages, more confidently to caregivers. There is room for improvement in how LMs are recruited, trained and supervised. The sustainability of the LM approach is at risk if policymakers do not find a way of transitioning the role of LMs into existing community health worker infrastructure and ensuring less reliance on donor support. Finally, the various factors that affect the role of LMs should be addressed and areas for improvement suggested by stakeholders should be given serious consideration as part of efforts to optimize the role of lead mothers during SMC campaigns and to improve health outcomes for SMC eligible children under 5 years.

## Data Availability

The dataset used and/or analysed during the current study are available from the corresponding author on reasonable request.

## Abbreviations

CDD: Community drug distributors
CHIPS: Community Health Influencers Promoters Services
CHWs: Community health workers
IEC: Information, education and communication
ITN: Insecticide-treated net
IPC: Infection prevention control
KSPHCMB: Kano state Primary Health Care Management Board
LGA: Local Government Area
LM: Lead mother
NMEP: National Malaria Elimination Programme
NPHCDA: National Primary Health Development Agency
SMC: Seasonal malaria chemoprevention
SPAQ: Sulfadoxine–pyrimethamine and amodiaquine
WDC: Ward Development Committee
WHO: World Health Organization
